# Aluminium Uptake and Translocation in Al Hyperaccumulator *Rumex obtusifolius* Is Affected by Low-Molecular-Weight Organic Acids Content and Soil pH

**DOI:** 10.1371/journal.pone.0123351

**Published:** 2015-04-16

**Authors:** Stanislava Vondráčková, Jiřina Száková, Ondřej Drábek, Václav Tejnecký, Michal Hejcman, Vladimíra Müllerová, Pavel Tlustoš

**Affiliations:** 1 Department of Agroenvironmental Chemistry and Plant Nutrition, Faculty of Agrobiology, Food and Natural Resources, Czech University of Life Sciences, Kamýcká 129, 165 21 Prague 6, Suchdol, Czech Republic; 2 Department of Soil Science and Soil Protection, Faculty of Agrobiology, Food and Natural Resources, Czech University of Life Sciences, Kamýcká 129, 165 21 Prague 6, Suchdol, Czech Republic; 3 Department of Ecology, Faculty of Environmental Sciences, Czech University of Life Sciences, Kamýcká 129, 165 21 Prague 6, Suchdol, Czech Republic; Universidade Federal de Vicosa, BRAZIL

## Abstract

**Background and Aims:**

High Al resistance of *Rumex obtusifolius* together with its ability to accumulate Al has never been studied in weakly acidic conditions (pH > 5.8) and is not sufficiently described in real soil conditions. The potential elucidation of the role of organic acids in plant can explain the Al tolerance mechanism.

**Methods:**

We established a pot experiment with *R*. *obtusifolius* planted in slightly acidic and alkaline soils. For the manipulation of Al availability, both soils were untreated and treated by lime and superphosphate. We determined mobile Al concentrations in soils and concentrations of Al and organic acids in organs.

**Results:**

Al availability correlated positively to the extraction of organic acids (citric acid < oxalic acid) in soils. Monovalent Al cations were the most abundant mobile Al forms with positive charge in soils. Liming and superphosphate application were ambiguous measures for changing Al mobility in soils. Elevated transport of total Al from belowground organs into leaves was recorded in both lime-treated soils and in superphosphate-treated alkaline soil as a result of sufficient amount of Ca available from soil solution as well as from superphosphate that can probably modify distribution of total Al in *R*. *obtusifolius* as a representative of “oxalate plants.” The highest concentrations of Al and organic acids were recorded in the leaves, followed by the stem and belowground organ infusions.

**Conclusions:**

In alkaline soil, *R*. *obtusifolius* is an Al-hyperaccumulator with the highest concentrations of oxalate in leaves, of malate in stems, and of citrate in belowground organs. These organic acids form strong complexes with Al that can play a key role in internal Al tolerance but the used methods did not allow us to distinguish the proportion of total Al-organic complexes to the free organic acids.

## Introduction

Accumulation of Al by plants has been seldom studied in weakly acidic soils [1–4]. It is known that uptake of Al by plants is affected by its mobile, potentially bioavailable forms in soils [4–6]. Mobile forms of metals are often referred to as exchangeable, water-soluble, and carbonate bound fractions [4,5]. Exudation of low-molecular-weight organic acids (LMWOAs; mainly citric, malic, and oxalic acids) commonly identified in the root zone can also play an important role in the uptake of Al by plants [7–9]. These natural organic substances are able to release metals from the exchangeable, carbonate, and reducible fractions and form soluble metal-organic acid complexes that are available to plants [10–13].

Many studies have investigated plants with mechanisms to tolerate high concentrations of Al, but usually in acidic soils [14,15]. Phytotoxicity of Al depends primarily on its chemical forms and not on the total accumulated amount in plants; not all species of mobile Al present an equally high toxicity to plants [3,9,16]. Aluminium toxicity to plants decreases in the order: polymer Al_13_ (not in a form of phosphates or silicates) > Al^3+^ > Al(OH)^2+^ > Al(OH)^+^_2_ > Al(OH)^-^_4_. Less or non-toxic Al species are supposed to be bound in sulphate, phosphate, silicate, fluoride or organic acids and Al(OH)_3_^0^ [17–21]. Chemical forms of Al are affected by soil pH [3,4,6,19]. Organic acids play a key role in Al tolerance mechanisms in plants; the type of organic acids and the secretion pattern depends on plant species [14,17,22–24]; distribution of organic acids between plant organs can be also crucial. Some plants detoxify Al externally, in the rhizosphere by releasing organic acids that chelate Al (i.e., *Triticum aestivum*, *Zea mays*, *Fagopyrum esculentum* or *Nicotiana tabacum*). Other plants, including species that accumulate Al in their leaves, detoxify Al internally by forming complexes with organic acids (i.e., *Camellia sinensis*, *Fagopyrum esculentum*, *Hydrangea* spp., *Melastoma malabathricum* or *Vaccinium macrocarpon*) [7,25–27].

*Rumex obtusifolius* subsp. *obtusifolius* (broad-leaved dock) is an important model plant with several specificities: (i) a widespread weedy species on arable land and in temperate grasslands [28], (ii) an oxalate plant with internal defence mechanism against Ca excess [29–30], (iii) an As-, Cd-, Pb-, and Zn-excluder not suitable for phytoremediation of heavily contaminated soils [31], and (iv) highly resistant to Al but the mechanism has been investigated only in hydroponics under strongly acidic conditions [30]. The high Al resistance of *R*. *obtusifolius* together with its ability to accumulate (>1000 mg/kg) [32] or even hyper-accumulate (>3000 mg/kg) [33] Al was not sufficiently described and explained in real soil conditions. The potential elucidation of the role of organic acids in plant organs can help to explain the mechanism of Al tolerance. The distribution of organic acids between aboveground organs in *Rumex* species has been studied [30,34] but insufficient information is available concerning the composition of organic acids in its belowground organs that are responsible for soil-plant interactions.

During our previous research [28,31], we found specific behaviour of Al in *R*. *obtusifolius* growing in tested soils (i.e. extremely high total concentration of Al in biomass compare to common plants). Therefore, we focused present study on the Al issue in this plant-soil interaction. The aim of this paper was to investigate the effect of slightly acidic and alkaline soils untreated and treated by lime and superphosphate on 1) concentration of mobile forms of Al as determined by mild soil extractants (KCl, CaCl_2_, and H_2_O) and by organic acids identified in root exudates (AA—acetic acid, CA—citric acid, and OA—oxalic acid), 2) concentration of individual positively charged Al species (Al(X)^1+^, Al(Y)^2+^, and Al^3+^) presented in exchangeable (KCl) and water-soluble (H_2_O) fraction, and on 3) distribution of total and infusion Al as well as of LMWOAs between organs of *R*. *obtusifolius*.

## Materials and Methods

### Pot Experiment

Two long-term heavily anthropogenically contaminated soils were used for the pot experiment in present study as well as in previous studies [28,31,35] with main chemical properties; ‘Litavka Fluvisol (49°43'N, 14°0'E)’ containing 354 mg As_*AR*_/kg, 54 mg Cd_*AR*_/kg, 3305 mg Pb_*AR*_/kg, and 6172 mg Zn_*AR*_/kg; characterised by pH_CaCl2_ 6.5, CEC 109 mmol_(+)_/kg, and C_org_ 3.6% and ‘Malín Luvisol (49°58´N, 15°17´E)’ containing 688 mg As_*AR*_/kg, 11 mg Cd_*AR*_/kg, and 1022 mg Zn_*AR*_/kg; characterised by pH_CaCl2_ 7.3, CEC 333 mmol_(+)_/kg, and C_org_ 2.7%. Litavka soil (forest soil) was sampled from the Ah horizon (0–15 cm) after removing the greensward layer; Malín soil (common arable soil) was sampled from the topsoil in the layer at 0–25 cm depth after removing the greensward layer.

The availability of Al in slightly acidic ‘Litavka’ and alkaline ‘Malín’ soils, which were both untreated and treated, was manipulated by lime and superphosphate application. We applied 7.3 g lime (CaO) per 1 kg of soil containing 686 g Ca/kg of material with pH_CaCl2_ 12.0 and 1.3 g superphosphate [Ca(H_2_PO_4_)_2_ H_2_O] per 1 kg of soil containing 246 g P/kg and 159 g Ca/kg of material with pH_CaCl2_ 2.2. The pot experiment was established in May 2011 with six treatments each with five replications: LC—Litavka control soil without any additive, LCa—Litavka soil with lime, LP—Litavka soil with superphosphate, MC—Malín control soil without any additive, MCa—Malín soil with lime, and MP—Malín soil with superphosphate. Five kg of air dried soil was passed through a 10 mm sieve then transferred to 5-L plastic pots (20 cm in diameter and height). In each pot, the whole soil profile was mixed with nutrient solution, consisting of 0.5 g N as NH_4_NO_3_, 0.16 g P and 0.4 g K as K_2_HPO_4_. Application of nutrient solution was performed, to ensure that N, P, and K availability was non-limiting for the growth of *R*. *obtusifolius* in all treatments. The lime and superphosphate additives were mixed with the soil after application of nutrient solution.

*Rumex obtusifolius* plants were grown in the pots for six months. The pots were regularly watered with deionised water to maintain the optimal moisture conditions for plant growth during the vegetation. At the harvest, plant biomass (3 plants per pot) was divided into belowground organs, stems, leaves, and seeds (i.e., achenes with a perianth) and subsequently soil samples were collected from the whole soil profile of each pot.

### Soil Analysis

For all chemical analyses, soil samples were air dried at 25°C and sieved to ≤2 mm. Before establishment of the pot experiment basic parameters of the experimental soils were determined by commonly used methods; microwave assisted high pressure *Aqua regia* (*AR*)-digestion (Ethos 1, MLS GmbH, Germany) for the determination of pseudo-total concentration of Al in soils by means of inductively coupled plasma-optical emission spectrometry (ICP-OES, VARIAN Vista Pro, Varian, Australia), soil pH in a 1/5 (w/v) suspension of soil and 0.01 mol/L CaCl_2_, cation-exchange capacity (CEC) was determined according to Schwertfeger and Hendershot [36], and organic carbon content (C_org_) colorimetrically according to Sims and Haby [37].

At the end of the experiment, soil samples were subjected to extraction with 0.5 mol/L KCl (adjusted to pH 5.8 by dilute HCl and KOH solutions; to extract the exchangeable fraction), 0.01 mol/L CaCl_2_ (pH 5.9; exchangeable fraction), and deionised water (H_2_O; pH 5.2; water-soluble fraction) in ratios 1/10 (w/v) [5,38] and by 0.11 mol/L acetic acid (AA; pH 2.8; exchangeable and carbonate fractions) [12,39], 0.11 mol/L citric acid (CA; pH 1.9; exchangeable, carbonate, and reducible fractions) [10,13], and 0.11 mol/L oxalic acid (OA; pH 1.3; exchangeable, carbonate, and reducible fractions) [10,13] in ratios 1/20 (w/v). The final concentration of AA, CA, and OA solutions was not realistic. The total concentration of Al in soil extracts (Al_KCl_, Al_CaCl2_, Al_H2O_, Al_AA_, Al_CA_, and Al_OA_) was determined using ICP-OES under standard conditions. Soil pH was measured in a suspension of soil and 0.01 mol/L CaCl_2_ (1/5, w/v) and 0.11 mol/L AA, CA, and OA (1/20, w/v). Detailed speciation of the exchangeable (KCl) and water-soluble forms of Al [species: Al(X)^1+^, Al(Y)^2+^, Al^3+^; and the sum of all forms of Al, Σ Al] according to the value of their positive charge was done by means of high performance liquid chromatography equipped with an ion column (HPLC/IC, Dionex, USA) [40]. Before analysis, samples were centrifuged and filtered using a 0.45-μm nylon membrane filter (Cronus Membrane Filter Nylon, GB) [5].

### Plant Analysis

The total concentration of Al in organs (Al/total; air-dried at 60°C and stainless-steel milled) was determined by ICP-OES after microwave assisted high pressure acid-digestion (65% HNO_3_:30% H_2_O_2_ 4:1). Certified reference material (CTA-OTL-1 oriental tobacco leaves) was mineralised under the same conditions for quality assurance. The concentration of Al in organ infusions (Al/infusion) was determined by ICP-OES after leaching (15 min) and filtering of the suspension of organ biomass and boiled deionised water (1/50, w/v) [41] through filtration paper ‘Filtrak 390’ (Niederschlag, Germany) with porosity 3–5 μm and flow rate 0.1 ml/s [DIN 53137]. After filtration through a 0.45-μm nylon membrane filter, concentrations of low-molecular-weight organic acid (LMWOAs; acetate, citrate, formate, lactate, malate, maleate, propionate, tartrate, and oxalate) anions in the same organ infusions were determined by means of ion-exchange chromatography with suppressed conductivity. An ion chromatograph ICS 1600 (Dionex, USA) equipped with IonPac AS11-HC (Dionex, USA) guard and analytical columns was used. The eluent composition was 1–37.5 mM KOH with a gradient of 1–50 min; and flow rate was set to 1 mL/min. To suppress eluent conductivity an ASRS 300–4 mm suppressor (Dionex, USA) and Carbonate Removal Device 200 (Dionex, USA) were used.

### Statistical Analysis

The statistical analysis was performed using Statistica 12.0 software (www.statsoft.com). All data were checked for homogeneity of variance and normality (Levene and Shapiro-Wilk tests). Soil and biomass data did not meet assumptions for the use of ANOVA and thus were evaluated by the non-parametric Kruskal-Wallis test. We assessed the effects of 1) treatment on soil pH, concentrations of Al in the soil and biomass and on concentration of LMWOAs in the biomass, 2) method of determination on concentration of Al, and 3) organ type on concentrations of Al and LMWOAs in the biomass. After obtaining significant results from the Kruskal-Wallis test, we used multiple comparisons of mean ranks for the detection of significant differences between different treatments or organs. The relationship between concentrations of 1) Al in different soil extracts, 2) Al/total (Al/infusion) in organs and Al in different soil extracts, and 3) Al/infusion and LMWOAs in organs was analysed by linear regression. A principal component analysis (PCA), in the CANOCO 4.5 program [42], was applied to all collected data together 1) concentration of Al in the soil extracts and 2) concentrations of Al/total, Al/infusion, and LMWOAs in the biomass of organs. We used standardisation of species data because data of different character were analysed together. The PCA was used to make visible correlations between all analysed data and similarity of different treatments. Obtained results were visualised in the form of a bi-plot ordination diagram in CanoDraw program.

## Results

### Fractionation of Al Using Soil Extractants

The concentration of Al extracted by *AR*, CaCl_2_, H_2_O, AA, CA, and OA was significantly affected by tested soil as well as by applied treatments (see Table 1 for details).

**Table 1:** Effect of treatment on soil pH (CaCl2, AA, CA, and OA), pseudo-total (mg/kg; extracted by *Aqua regia*; *AR*), exchangeable (mg/kg; extracted by 0.5 mol/L KCl or by 0.01 mol/L CaCl2), water-soluble (mg/kg; extracted by deionised water; H2O), exchangeable and carbonate (mg/kg; extracted by 0.11 mol/L acetic acid; AA), and exchangeable, carbonate, and reducible (mg/kg; extracted by 0.11 mol/L citric acid; CA or by 0.11 mol/L oxalic acid; OA) concentration of Al (mean ± SE) at the end of the experiment.

Variable	Treatment					
	LC	LCa	LP	MC	MCa	MP
pH_CaCl2_ **	5.8^b^ ± 0.01	7.5^a^ ± 0.01	5.9^b^ ± 0.02	7.2^ab^ ± 0.03	7.6^a^ ± 0.02	7.2^ab^ ± 0.01
pH_AA_ **	3.9^b^ ± 0.01	4.3^ab^ ± 0.03	3.9^b^ ± 0.003	4.6^ab^ ± 0.03	5.0^a^ ± 0.02	4.6^ab^ ± 0.01
pH_CA_ **	2.3^b^ ± 0.01	2.5^abc^ ± 0.02	2.3^bc^ ± 0.01	2.5^ac^ ± 0.01	2.7^a^ ± 0.02	2.5^ac^ ± 0.02
pH_OA_ **	1.4^b^ ± 0.01	1.4^ab^ ± 0.01	1.4^b^ ± 0.02	1.4^ab^ ± 0.01	1.4^a^ ± 0.01	1.4^ab^ ± 0.01
Al_*AR*_ *	11067^b^ ± 317	-	-	13482^a^ ± 595	-	-
Al_KCl_^n.s.^	11^a^ ± 0.1	9^a^ ± 0.1	10^a^ ± 2	10^a^ ± 1	12^a^ ± 1	11^a^ ± 1
Al_CaCl2_ *	12^ab^ ± 1	9^b^ ± 1	15^ab^ ± 3	11^ab^ ± 1	18^a^ ± 1	13^ab^ ± 1
Al_H2O_ **	81^ab^ ± 3	70^abc^ ± 2	106^a^ ± 5	22^bc^ ± 1	15^c^ ± 1	21^bc^ ± 1
Al_AA_ *	113^a^ ± 11	102^ab^ ± 21	86^ab^ ± 8	59^a^ ± 3	80^ab^ ± 4	69^ab^ ± 6
Al_CA_ **	1188^ab^ ± 9	1197^ab^ ± 7	1149^b^ ± 5	1968^a^ ± 8	1896^ab^ ± 2	1974^a^ ± 4
Al_OA_ **	3070^ab^ ± 5	2940^b^ ± 42	2972^b^ ± 17	5119^a^ ± 35	4908^ab^ ± 8	5052^a^ ± 25

– indicates not determined

Treatment abbreviations: LC—Litavka control soil without any additive, LCa—Litavka soil with lime, LP—Litavka soil with superphosphate, MC—Malín control soil without any additive, MCa—Malín soil with lime, and MP—Malín soil with superphosphate.

Calculated by Kruskal-Wallis test, differences between treatments were not statistically significant (^n.s.^) or were significant at 0.05 (*) and 0.01 (**) probability levels. According to the multiple comparisons of mean ranks, treatments with the same letter were not significantly different.

**Fig 1:**
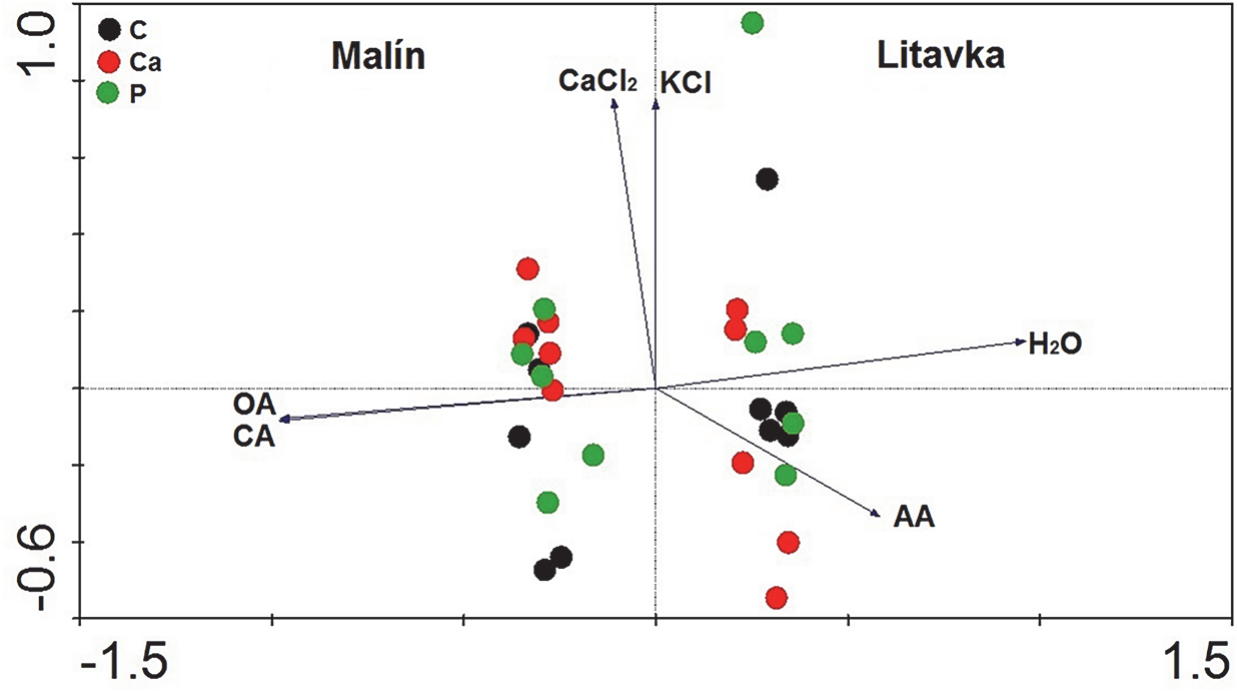
Ordination diagram showing the results of PCA analysis with total concentration of Al in soil extracts in contaminated slightly acidic Litavka and alkaline Malín soils. Treatment abbreviations: C—control, Ca—lime application, P—superphosphate application. Soil extractant abbreviations: KCl—exchangeable concentration of Al in soil (extracted by 0.5 mol/L KCl), CaCl_2_—exchangeable concentration of Al in soil (extracted by 0.01 mol/L CaCl_2_), H_2_O —water-soluble concentration of Al in soil (extracted by deionised water), AA—exchangeable and carbonate concentration of Al in soil (extracted by 0.11 mol/L acetic acid), CA—exchangeable, carbonate, and reducible concentration of Al in soil (extracted by 0.11 mol/L citric acid), and OA—exchangeable, carbonate, and reducible concentration of Al in soil (extracted by 0.11 mol/L oxalic acid).

The efficiency of KCl and CaCl_2_ for extractability of Al was comparable in both soils (LC, LCa, LP, MC, MCa, and MP treatments). Higher efficiency of H_2_O and AA for Al extractability was recorded in Litavka soil (LC, LCa, and LP treatments) and of CA and OA was recorded in Malín soil (MC, MCa, and MP treatments; see Fig 1). A significant negative relationship was recorded between the concentration of Al extracted by H_2_O and CA (r = –0.947; p<0.01), by H_2_O and OA (r = –0.944; p<0.01), by AA and CA (r = –0.549; p<0.01), and by AA and OA (r = –0.562; p<0.01). A significant positive relationship was recorded between concentrations of Al extracted by H_2_O and AA (r = 0.408; p = 0.043) and by CA and OA (r = 0.997; p<0.01).

Liming (LCa and MCa treatments) and application of superphosphate (LP and MP treatments) did not significantly affect the concentration of Al in soils.

### Speciation of Exchangeable (KCl) and Water-Soluble Forms of Al in the Soil

**Fig 2:**
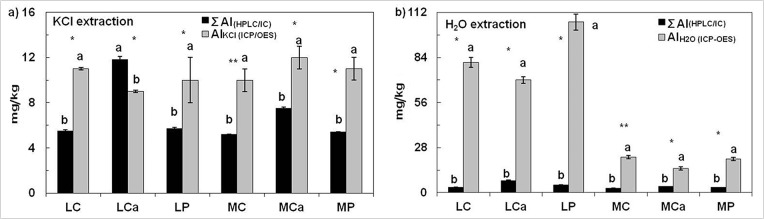
Effect of method of determination (HPLC/IC and ICP-OES) on mean concentration of exchangeable and water-soluble forms of Al (total AlKCl and total AlH2O; and Σ Al, all Al forms positively charged) at the end of the experiment. Treatment abbreviations: LC—Litavka control soil without any additive, LCa—Litavka soil with lime, LP—Litavka soil with superphosphate, MC—Malín control soil without any additive, MCa—Malín soil with lime, and MP—Malín soil with superphosphate. Error bars represent SE. Based on Kruskal-Wallis test, differences between methods of determination were significant at 0.05 (*) and 0.01 (**) probability levels. Using multiple comparisons of mean ranks, methods of determination with the same letter were not significantly different.

The difference between concentrations of Σ Al and Al_KCl_ (Al_H2O_) was significantly affected by the method of determination in all treatments (see Fig 2 for details).

Exchangeable and water-soluble concentrations of Al(X)^1+^, Σ Al and water-soluble concentration of Al(Y)^2+^ were significantly affected by treatments (see Table 2 for details).

**Table 2:** Effect of treatment on exchangeable and water-soluble forms of Al (mg/kg; species: Al(X)1+, Al(Y)2+, Al3+; and Σ Al—all Al forms positively charged; total AlKCl and total AlH2O; mean ± SE) at the end of the experiment.

Extracting reagent	Variable	Treatment					
		LC	LCa	LP	MC	MCa	MP
KCl	Al(X)^1+^ **	5.5^abc^ ± 0.1	11.8^a^ ± 0.3	5.7^abc^ ± 0.1	5.2^c^ ± 0.04	7.5^ab^ ± 0.1	5.4^c^ ± 0.05
	Al(Y)^2+^	<0.5	<0.5	<0.5	<0.5	<0.5	<0.5
	Al^3+^	<0.5	<0.5	<0.5	<0.5	<0.5	<0.5
	Σ Al **	5.5^abc^ ± 0.1	11.8^a^ ± 0.3	5.7^abc^ ± 0.1	5.2^c^ ± 0.04	7.5^ab^ ± 0.1	5.4^c^ ± 0.05
	Al_KCl_^n.s.^	11^a^ ± 0.1	9^a^ ± 0.1	10^a^ ± 2	10^a^ ± 1	12^a^ ± 1	11^a^ ± 1
H_2_O	Al(X)^1+^ **	3.3^ab^ ± 0.1	5.8^a^ ± 0.3	3.5^ab^ ± 0.1	2.8^b^ ± 0.1	4.0^a^ ± 0.04	3.2^ab^ ± 0.1
	Al(Y)^2+^ *	<0.5	0.9^a^ ± 0.1	0.6^b^ ± 0.05	<0.5	<0.5	<0.5
	Al^3+ n.s.^	<0.5	0.7^a^ ± 0.1	0.6^a^ ± 0.1	<0.5	<0.5	<0.5
	Σ Al **	3.3^abc^ ± 0.1	7.4^a^ ± 0.4	4.8^ab^ ± 0.1	2.8^c^ ± 0.05	4.0^abc^ ± 0.04	3.2^bc^ ± 0.1
	Al_H2O_ **	81^ab^ ± 3	70^abc^ ± 2	106^a^ ± 5	22^bc^ ± 1	15^c^ ± 1	21^bc^ ± 1

limit of detection (mg/kg): 0.5

Treatment abbreviations: LC—Litavka control soil without any additive, LCa—Litavka soil with lime, LP—Litavka soil with superphosphate, MC—Malín control soil without any additive, MCa—Malín soil with lime, and MP—Malín soil with superphosphate.

Calculated by Kruskal-Wallis test, differences between treatments were not statistically significant (^n.s.^) or were significant at 0.05 (*) and 0.01 (**) probability levels. According to the multiple comparisons of mean ranks, treatments with the same letter were not significantly different.

Liming (LCa and MCa treatments) increased the exchangeable and water-soluble concentrations of Al(X)^1+^ in both soils (LC and MC treatments). Concentrations of exchangeable Al(Y)^2+^ and Al^3+^ were below the limit of detection (0.5 mg Al/kg) in all treatments. Concentrations of water-soluble Al(Y)^2+^ and Al^3+^ were below the limit of detection (0.5 mg Al/kg) in most of the treatments (LC, MC, MCa, and MP). Liming (LCa treatment) and application of superphosphate (LP treatment) increased the water-soluble concentrations of Al(Y)^2+^ and Al^3+^ in soil (LC treatment).

### Concentration of Al in the Plant Organs

The Al/total concentration was significantly affected by treatments and analysed plant organs (see Table 3 for details). The concentration of Al/total in seeds ranged from 51±19 mg/kg in LCa treatment to 718±213 mg/kg in MC treatment. No stems or seeds were produced in the LC and LP treatments.

**Table 3:** Effect of treatment on concentration of total and infusion Al (mg/kgDW; mean ± SE) in organs of *R*. *obtusifolius*.

Variable	Treatment	Organ		
		Belowground organs	Stems	Leaves
Al/total	LC	1702^aA^ ± 604	-	471^aBC^ ± 76
deficient	LCa	693^abA^ ± 344	41^bB^ ± 9	933^aABC^ ± 244
normal <100–200^1^	LP	1052^aA^ ± 433	-	384^aC^ ± 88
phytotoxic	MC	3514^abA^ ± 1583	169^bAB^ ± 63	3413^aAB^ ± 794
accumulation level >1000^1^	MCa	584^abA^ ± 257	133^bAB^ ± 49	1772^aABC^ ± 528
hyperaccumulation level >3000^2^	MP	1767^abA^ ± 792	198^bA^ ± 25	3858^aA^ ± 794
Al/infusion	LC	10.2^A^	-	-
	LCa	4.9^abA^ ± 0.5	1.2^bA^ ± 0.4	93^aA^ ± 40
	LP	6.1^aA^ ± 0.6	-	6.2^aA^ ± 2.6
	MC	5.9^bA^ ± 2.4	4.1^abA^	75^aA^ ± 19
	MCa	3.6^bA^ ± 0.5	6.3^abA^ ± 3.5	38^aA^ ± 14
	MP	5.9^aA^ ± 1.3	-	97^bA^ ± 21

References: 1– [32], 2– [33]

—indicates no material

Treatment abbreviations: LC—Litavka control soil without any additive, LCa—Litavka soil with lime, LP—Litavka soil with superphosphate, MC—Malín control soil without any additive, MCa—Malín soil with lime, and MP—Malín soil with superphosphate.

Differences between treatments and organs were evaluated by Kruskal-Wallis test. For each element, concentrations in organs within one treatment denoted with the same letter (a-b) and concentrations in treatments within one organ denoted with the same letter (A-C) were not significantly different.

A tendency for higher Al/total concentration in organs was recorded in Malín soil (MC, MCa, and MP treatments) in comparison to Litavka soil (LC, LCa, and LP treatments).

A significant positive relationship was recorded between Al/total concentration in leaves and Al_OA_ concentration in soil (all treatments all together; r = 0.756, p<0.01), and between Al/total concentration in organs and Al_CA_ concentration in soil (all treatments all together; stems: r = 0.652, p = 0.012; leaves: r = 0.717, p<0.01; seeds: r = 0.560, p = 0.047).

Liming (LCa and MCa treatments) and application of superphosphate (MP treatment) affected the distribution of Al/total concentration between organs in the order: belowground organs < leaves in comparison to LC and MC treatments (belowground organs > leaves).

### Concentrations of LMWOAs in the Organs

The concentrations of propionate, malate, maleate, and citrate were significantly affected by treatments, and the concentrations of acetate, citrate, formate, lactate, malate, maleate, propionate, tartrate, and oxalate differed between individual organs (see Table 4 for details).

**Table 4:** Effect of treatment on concentrations of LMWOAs (mg/kgDW, mean ± SE) in organs of *R*. *obtusifolius*.

Variable	Treatment	Organ		
		Belowground organs	Stems	Leaves
Acetate	LC	817^A^	-	-
	LCa	674^aA^ ± 6	614^aA^ ± 63	2028^aA^ ± 491
	LP	629^aA^ ± 47	-	641^aA^ ± 14
	MC	623^bA^ ± 96	1089^abA^	1898^aA^ ± 368
	MCa	643^aA^ ± 60	569^aA^ ± 7	1852^aA^ ± 11
	MP	662^aA^ ± 52	-	1887^aA^ ± 493
Citrate	LC	8825^AB^	-	-
	LCa	5845^aAB^ ± 786	2028^aA^ ± 858	5629^aA^ ± 806
	LP	7939^aA^ ± 886	-	6754^aA^ ± 347
	MC	6068^aAB^ ± 522	2094^aA^	4275^aA^ ± 630
	MCa	5838^aAB^ ± 307	2130^bA^ ± 582	4742^abA^ ± 550
	MP	5202^aB^ ± 151	-	3875^bA^ ± 487
Formate	LC	244^AB^	-	-
	LCa	184^bAB^ ± 20	238^abA^ ± 22	328^aAB^ ± 9
	LP	153^aAB^ ± 12	-	267^aB^ ± 19
	MC	251^aA^ ± 33	418^aA^	362^aAB^ ± 27
	MCa	89^bB^ ± 8	218^abA^ ± 4	443^aA^ ± 45
	MP	204^bAB^ ± 38	-	367^aAB^ ± 6
Lactate	LC	744^A^	-	-
	LCa	914^bA^ ± 113	1303^abA^ ± 70	5982^aA^ ± 1398
	LP	646^aA^ ± 70	-	2424^aA^ ± 40
	MC	733^bA^ ± 14	1615^abA^	6442^aA^ ± 812
	MCa	707^bA^ ± 57	1587^abA^ ± 226	4081^aA^ ± 956
	MP	723^bA^ ± 25	-	6385^aA^ ± 466
Malate	LC	2429^AB^	-	-
	LCa	2951^bA^ ± 213	6504^aA^ ± 340	3751^abAB^ ± 125
	LP	1877^aB^ ± 115	-	2996^aAB^ ± 508
	MC	2290^aAB^ ± 72	8769^aA^	2821^aAB^ ± 245
	MCa	2464^bAB^ ± 292	6679^aA^ ± 1582	4527^aA^ ± 487
	MP	2408^aAB^ ± 240	-	2197^aB^ ± 487
Maleate	LC	88^AB^	-	-
	LCa	139^aA^ ± 19	16^abA^ ± 4	<2.43^bA^
	LP	104^aAB^ ± 14	-	<2.43^aA^
	MC	<2.43^aB^	<2.43^aA^	<2.43^aA^
	MCa	77^aAB^ ± 25	17^aA^ ± 10	12^aA^ ± 7
	MP	<2.43^aAB^	-	12^aA^ ± 6
Propionate	LC	<1.94^A^	-	-
	LCa	<1.94^aA^	<1.94^aA^	91^aAB^ ± 57
	LP	48^aA^ ± 20	-	363^aAB^ ± 152
	MC	<1.94^aA^	132^aA^	<1.94^aAB^
	MCa	<1.94^bA^	<1.94^abA^	407^aA^ ± 52
	MP	<1.94^aA^	-	<1.94^aB^
Tartrate	LC	6245^A^	-	-
	LCa	5166^aA^ ± 335	659^bA^ ± 33	2324^abB^ ± 341
	LP	5362^aA^ ± 211	-	4063^aAB^ ± 372
	MC	5313^aA^ ± 230	1008^aA^	4168^aAB^ ± 660
	MCa	5419^aA^ ± 255	550^bA^ ± 12	3076^abAB^ ± 111
	MP	4569^aA^ ± 85	-	4081^bA^ ± 165
Oxalate	LC	1510^A^	-	-
	LCa	1355^bA^ ± 133	4427^abA^ ± 699	11019^aA^ ± 95
	LP	1401^aA^ ± 61	-	10884^aA^ ± 212
	MC	1570^bA^ ± 171	8449^abA^	14594^aA^ ± 543
	MCa	1494^bA^ ± 45	5486^abA^ ± 158	13234^aA^ ± 1701
	MP	1569^bA^ ± 67	-	11180^aA^ ± 765

– indicates no material, limits of detection (mg/kg): acetate 1.37, citrate 3.18, formate 0.61, lactate 1.13, malate 1.85, maleate 2.43, propionate 1.94, tartrate 1.73, and oxalate 1.08.

Treatment abbreviations: LC—Litavka control soil without any additive, LCa—Litavka soil with lime, LP—Litavka soil with superphosphate, MC—Malín control soil without any additive, MCa—Malín soil with lime, and MP—Malín soil with superphosphate.

Differences between treatments and organs were evaluated by Kruskal-Wallis test. For each LMWOA, concentrations in organs within one treatment denoted with the same letter (a-b) and concentrations in treatments within one organ denoted with the same letter (A-B) were not significantly different.

In all treatments, a tendency for higher accumulation of citrate, maleate, and tartrate was recorded in belowground organs; a tendency for higher accumulation of malate was recorded in stems; and a tendency for higher accumulation of acetate, formate, lactate, propionate, and oxalate was recorded in leaves.

The distribution of total concentration of LMWOAs (65426 mg/kgDW, i.e., sum of the mean value of all organic acids concentration in the organs) between organs was recorded in the order: belowground organs (17091 mg/kgDW; 26% of total LMWOAs) < stems (17951 mg/ kgDW; 27% of total LMWOAs) < leaves (30384 mg/kgDW; 47% of total LMWOAs). Representation of LMWOAs in belowground organs was in the order: citrate (36%) > tartrate (31%) > malate (14%) > oxalate (9%) > acetate, lactate (4%) > formate (1%) > maleate (0.4%) > propionate (0.05%); in stems was in order malate (39%) > oxalate (32%) > citrate (12%) > lactate (8%) > acetate, tartrate (4%) > formate (1.5%) > propionate (0.3%) > maleate (0.1%); and in leaves was in the order: oxalate (39%) > lactate (17%) > citrate (15%) > malate, tartrate (11%) > acetate (6%) > formate (1%) > propionate (0.5%) > maleate (0.03%).

A significant positive relationship was recorded between concentrations of Al/infusion and LMWOAs in belowground organs (acetate: r = 0.670, p<0.01; formate = 0.574, p<0.01), between concentrations of Al/infusion and lactate in stems (r = 0.944, p = 0.016), and between concentrations of Al/infusion and LMWOAs in leaves (acetate: r = 0.695, p<0.01; lactate = 0.773, p<0.01).

Liming (LCa and MCa treatments) and application of superphosphate (LP and MP treatments) did not lead to the unambiguous changes in the distribution of LMWOAs between organs.

### Results of PCA Analysis

#### Soil

The first axis of the PCA analysis explained 53%, the first two axes 75% and the first four axes together, 99% of the variability of all analysed data (Fig 1). The first ordination axis divided marks for individual pots into Litavka group on the right side and Malín group on the left side of the diagram. This indicates a high effect of used soils on the extractability of Al. In both soils, marks for treatments (C, Ca, and P) were not clearly separated, which indicates minimal effect of treatments on all the recorded data. The length and direction of the vectors relating to the Al concentrations indicate the association of the extractants with respect of studied soil. Di- and tri-carboxylic acids such as OA and CA extracted more Al in alkaline Malín soil and mono-carboxylic acid AA and H_2_O extracted more Al in slightly acidic Litavka soil. Extractants KCl and CaCl_2_ had minimal effect on extractability of Al in both soils as shown by arrows not leading to any of the soils. Concentrations of Al_CA_ and Al_OA_ were negatively correlated with Al_H2O_ as indicated by opposing directions of vectors for CA (OA) and H_2_O.

##### Plant Biomass

**Fig 3:**
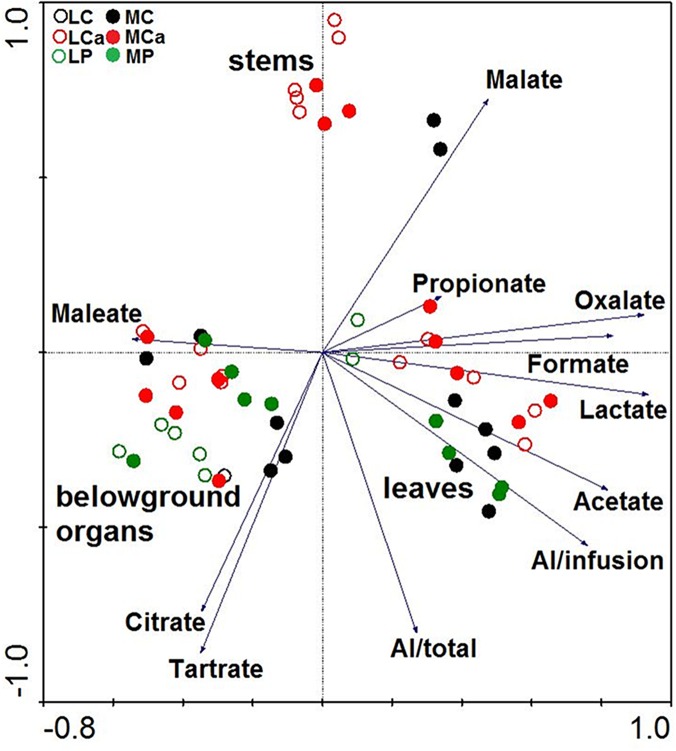
Ordination diagram showing the results of PCA analysis with concentrations of Al and of LMWOAs in organs (belowground organs, stems, and leaves) of *R*. *obtusifolius* grown on contaminated soils. Treatment abbreviations: LC—Litavka control soil without any additive, LCa—Litavka soil with lime, LP—Litavka soil with superphosphate, MC—Malín control soil without any additive, MCa—Malín soil with lime, and MP—Malín soil with superphosphate. LMWOAs abbreviations: acetate—concentration of acetate in organ infusions and etc. Al concentration abbreviations: Al/total—total concentration of Al in organs, Al/infusion—concentration of Al in organ infusions.

The first axis of the PCA analysis explained 42%, the first two axes 69% and the first four axes together, 86% of the variability of all analysed data (Fig 3). In the diagram, marks for treatments (LC, LCa, LP, MC, MCa, and MP) were not clearly separated indicating a weak effect of soils and treatments on all the recorded data. Marks for organs (belowground organs, stems, and leaves) were located in different parts of the diagram, which indicates a high effect of organs on all the recorded data. The length and direction of the vectors relating to the Al and LMWOAs concentrations indicate the association of the Al as well as LMWOAs with respect of organ. Concentrations of citrate, maleate, and tartrate were accumulated more in belowground organs. Concentrations of Al/infusion as well as concentrations of acetate, formate, lactate, propionate, and oxalate accumulated more in leaves and malate accumulated more in stems. Al/total accumulated more in belowground organs as well as in leaves.

## Discussion

### Fractionation of Al Using Soil Extractants

Efficiency of extracting agents in LC and MC treatments increased in the following order: Al_KCl_, Al_CaCl2_ < Al_H2O_ < Al_AA_ < Al_CA_ < Al_OA_ representing 0.1, 0.1 < 0.2–0.7 < 0.4–1.0 < 11–15 < 28–38% of pseudo-total soil Al (Al_*AR*_). Exchangeable (KCl, CaCl_2_), water-soluble (H_2_O), and carbonate (AA) bound fractions of Al do not present an important role in Al mobility in slightly acidic and alkaline soils. The highest portions of Al were released by extracting with di- and tri-carboxylic acids (OA and CA), commonly identified in root exudates, which can also release Al bound in the reducible fraction [8,10–13]. The efficiency of these LMWOAs in soil can be explained by their dissociation constants: OA: pK_a_ 1.25 (I), 4.26 (II); CA: pK_a_ 3.13 (I), pK_a_ 4.76 (II), pK_a_ 6.40 (III); AA: pK_a_ 4.76. The number of dissociation constants corresponds to the number of COOH groups; a lower value of the negative logarithm of the dissociation constant of acid—pK_a_ means a higher dissociation. i.e. A higher acid strength) [43] and therefore a decrease of soil pH (i.e., pH_AA_ < pH_CA_ < pH_OA_; Table 1). Thus, we observed that the ability of LMWOAs to release Al from reducible fraction increased in the order AA < CA < OA. We can conclude from our experiment that ‘exudation’ of organic acids by *R*. *obtusifolius* (mainly oxalic acid) significantly affected the mobility of Al in slightly acidic and alkaline soils and helped to make a real estimate of Al uptake [12,22,44,45].

Reagents such as KCl and CaCl_2_ were not suitable for the evaluation of available Al in all treatments. It is connected with the sorption complex with a dominant representation of the basic cations (Ca, Mg, and K) in comparison to the acidic cations (Al, Fe, and Mn) in slightly acidic and alkaline soils. Reagents such as H_2_O and AA showed higher efficiency for extractability of Al in LC, LCa, and LP treatments in comparison to MC, MCa, and MP treatments. It is connected also with the exchangeable fraction, which has greater importance for Al in slightly acidic soil (‘Litavka’) than in alkaline soil (‘Malín’). Deionised water and AA reagents with their lower solution pH (H_2_O 5.2 and AA 2.8) are able to release Al into soil solution with higher efficiency than KCl and CaCl_2_ reagents (KCl pH 5.8 and CaCl_2_ pH 5.9) because of low representation of Al on the sorption complex. Therefore, pH probably plays a key role rather than the exchange of Al for K or Ca ions. Moreover, deionised water has high extraction power due to low ionic strength [46]. The AA reagent probably showed greater importance in releasing Al from the exchangeable fraction than from the carbonate bound fraction. It is because more carbonates were determined in the MC treatment (above 2% of carbonates) in comparison to the LC treatment (below 0.3% of carbonates; data not shown). Nevertheless, higher efficiency of the AA reagent was observed in slightly acidic soil (LC, LCa, and LP treatments). We can speculate that carbonates do not present important binding sites for Al [9] as a sorption complex in slightly acidic soils. Reagents such as CA and OA have higher efficiency for extractability of Al in MC, MCa, and MP treatments in comparison to LC, LCa, and LP treatments. It is probably connected with increased root exudates efficiency for organic acids exclusion in alkaline soil conditions (MC treatment) in comparison to slightly acidic soil conditions (LC treatment) [15]. The effect of liming (LCa and MCa treatments) on increase of soil pH was neglected with increasing strength of studied agents (see pH_AA_, pH_CA_, and pH_OA_ in Table 1).

Liming and application of superphosphate has an ambiguous effect on the total extractable concentration of Al (i.e. Al_KCl_, Al_CaCl2_, Al_H2O_, Al_AA_, Al_CA_, and Al_OA_) in soil indicating that our tested additives are not able to significantly alter the mobility of Al neither in slightly acidic nor in alkaline soils.

### Speciation of Exchangeable (KCl) and Water-Soluble Forms of Al in the Soil

In most of the treatments, total extractable Al (i.e., Al_KCl_ and Al_H2O_) was determined to be higher than the Σ Al, indicating that the derivatization agent ‘Tiron (i.e., 4,5-dihydroxy-m-benzendisulfonic acid)’ used in HPLC/IC did not react with certain strongly complexed Al forms with presumably zero or negative charge. However, these Al species undetectable by HPLC/IC can be determined by ICP-OES [5].

The most abundant exchangeable and water-soluble Al forms with positive charge in LC and MC treatments were Al(X)^1+^ species (i.e., Al(OH)_2_^+^, Al(SO_4_)^+^, AlF_2_^+^, Al(org.)^≤1+^, etc.; [5,40] representing 51% and 8.5% of total Al_KCl_ and Al_H2O_, respectively. This can be explained by the distribution of soluble Al forms in the pH range between 5.8 and 7.2, with dominant representation of less or non-toxic Al forms with positive charge (Al(X)^1+^) as well as with zero (Al(OH)_3_^0^, Al-organic complex) and negative charge (Al(OH)_4_^-^, Al-organic complex) [3,4,6,19].

Liming (LCa and MCa treatments) increased the exchangeable and water-soluble Al(X)^1+^ form in comparison to the LC and MC treatments. Moreover, liming (LCa) and application of superphosphate (LP) caused the occurrence of new forms of Al such as Al(Y)^2+^ (1.3% and 0.6% of Al_H2O_, respectively) and Al^3+^ (1% and 0.6% of Al_H2O_, respectively) which were identified in the H_2_O-soil solution. It is because lime caused mineralisation of organic matter (representing an important binding site for Al) and decay of bound complexes to the individual forms of Al determinable using HPLC/IC [47,48]. In the case of superphosphate, the release of new forms of Al (i.e., Al(Y)^2+^ and Al^3+^) can be explained by a sufficient amount of available Ca from superphosphate that caused the decay of organic matter with lower stability.

### Concentration of Al in the Plant Organs

Concentration of Al/total in aboveground organs was clearly positively related to the concentrations of Al_CA_ and Al_OA_ in soil, indicating their important role in uptake of Al by plants. Therefore, we can conclude that the Al availability for *R*. *obtusifolius* has been more affected by the presence of root exudates releasing organic acids (mainly oxalic acid) than by ‘mobile forms’ of Al (i.e., exchangeable, water-soluble, and carbonate) in slightly acidic and alkaline soils. A considerable decrease of pH down to 2.3 (CA:soil solution) and to 1.4 (OA:soil solution) is connected with possible uptake of Al by plants in the form of Al^3+^, as has been recorded also by Ma et al. [7].

Higher concentration of Al/total in all organs was recorded in alkaline soil (MC treatment) in comparison to slightly acidic soil (LC treatment). It is probably due to a generally higher pseudo-total Al concentration in MC treatment (see Table 1) resulting in more available Al_CA_ and Al_OA_ present for plants, and in increased root exudates efficiency for organic acids exclusion in alkaline soil conditions than in slightly acidic soil conditions, as has been recorded by Arunakumara et al. [15].

A tendency for restricted transport of Al/total from belowground organs into the leaves was recorded in the LC treatment, in comparison to MC treatment. We can speculate that differences in the distribution can be connected with a different mechanism for detoxification of higher concentrations of Al/total in *R*. *obtusifolius* planted in slightly acidic (probably external detoxification of Al) and alkaline (probably internal detoxification of Al) soils. Low transport from belowground organ to leaf as a defence mechanism against high concentration of Al in plants was described in study of Poschenrieder et al. [21]. The effectiveness of both mechanisms for Al detoxification in the same plant is not unique (e.g. *Fagopyrum esculentum*) [49,50]. The mechanisms are known also for relatives to *R*. *obtusifolius*—*R*. *acetosella* (external) and *R*. *acetosa* (internal) planted in strongly acidic conditions [26,48].

*R*. *obtusifolius* is able to tolerate a high concentration of Al/total because in its leaves substantially higher Al concentration was recorded than is normal in many other plants (<200 mg/kg) in LC and MC treatments. In MC treatment, the concentration of Al/total in leaves was higher than 1000 mg/kg therefore we can speculate that *R*. *obtusifolius* belongs among the ‘Al accumulators’ or even the ‘Al hyper-accumulators’ (>3000 mg/kg) if planted in alkaline soils. High Al tolerance is probably connected with internal Al detoxification (formation of Al-complex, mainly with organic acids, in parts of leaves that are insensitive to Al, e.g., epidermal cells of vacuoles and cell walls) typical for ‘Al-accumulators’ but known mainly in acid soils [21,26,32,44,51,52].

In MC treatment, the concentration of Al/infusion in leaves was higher than in stems and belowground organs. In all treatments, higher leaching of Al from leaf and stem infusions (2–10% and 2–5% of Al/total, respectively) were recorded in comparison to belowground organ infusion (0.2–0.7% of Al/total), indicating higher representation of Al-organic complexes with weak stability constants [16,53] in aboveground organs. The concentration of Al leached from leaf infusion of *R*. *obtusifolius* (in most treatments—2% of total Al) was lower in comparison to plants commonly used as beverages (i.e., *Hibiscus sabdariffa* petals—50% of total Al, *Rosa canina* receptacles—30% of total Al, *Camellia sinensis* leaves—10% of total Al, *Cymbopogon citratus* leaves—8% of total Al or *Ginkgo biloba* leaves—4% of total Al) [41,54]. Nevertheless, potential risk of a harmful effect of Al for humans remains because of the use of *R*. *obtusifolius* as a component of salad and soup, rather than as a beverage [55,56].

Liming (LCa and MCa treatments) and application of superphosphate (MP treatment) caused increased transport of Al/total from belowground organs into leaves in comparison to LC and MC treatments. It is probably due to the presence of oxalate (*R*. *obtusifolius* belongs to the group of ‘oxalate plants’) [29] that was precipitated with Ca as Ca-oxalate and thus available Al can be easily transported to leaves in LCa, MCa, and MP treatments. In LC and MC treatments, Al was probably immobilized as Al-oxalate in belowground organs, as has been recorded also for micro- (Cu, Fe, Mn, Ni) and risk elements (As, Cd, Cr, Pb, Zn) by Vondráčková et al. [31]. Nevertheless, the used methods did not allow us to determine the specific metal-organic complexes in plants or to distinguish the proportion of total metal-organic complexes (e.g. metal-oxalate complex) to the free organic acids (e.g. oxalic acid). Therefore, we are not able to verify the above-mentioned mechanism.

### Concentrations of LMWOAs in the Organs

The LMWOAs were divided into three groups according to the detected concentration in organs. Citrate, maleate, and tartrate were recorded more in belowground organs. Malate was recorded more in stems, and acetate, formate, lactate, propionate, and oxalate were recorded more in leaves. The chemical structure of LMWOAs (position of OH/COOH groups on their main C chain) in connection with the stability constants (log*K*s) of Al-organic complexes can explain the ability of LMWOAs to create various strong complexes with Al in organs (higher value of the stability constant means higher stability of Al-organic complex) [16,43,53,57,58]. We can conclude that strong complexes of LMWOAs with Al (Al-citrate—log*K*s = 7.98 and Al-tartrate—log*K*s = 5.62) were recorded in belowground organs, moderate complexes of LMWOAs with Al (Al-malate—log*K*s = 5.40) were recorded in stems and weak complexes of LMWOAs with Al (Al-acetate—log*K*s = 1.60, Al-formate—log*K*s = 1.36, Al-lactate—log*K*s = 2.41, and Al-propionate—log*K*s = 1.78) were recorded in leaves. Nevertheless, two exceptions were recorded; Al-maleate complex with weak stability constant (log*K*s = 1.93) in belowground organs and Al-oxalate complex with strong stability constant (log*K*s = 6.16) in leaves. Concentration of Al in leaf infusions was clearly positively related to the concentrations of acetate (representing 6% of total LMWOAs) and lactate (17% of total LMWOAs); concentration of Al in stem infusions was clearly positively related to the concentration of lactate (8% of total LMWOAs); and concentration of Al in belowground organ infusions was clearly positively related to the concentrations of acetate (4% of total LMWOAs) and formate (1% of total LMWOAs), which indicates a weak stability complex of Al with these LMWOAs in organs. Therefore, possible release of Al into infusions was recorded with increasing order: belowground organs < stems < leaves.

The highest concentration of all LMWOAs all together was recorded in leaves, followed by stems and belowground organs. Oxalate, citrate, malate, and tartrate were dominant LMWOAs in leaves; malate, oxalate, and citrate were dominant LMWOAs in stems; and citrate, tartrate, and malate were dominant LMWOAs in belowground organs. Miyagi et al. [34] have recorded a similar result, i.e. oxalate as a major LMWOA in leaves of *R*. *obtusifolius* grown for a period of 2 and 5 weeks in a hydroponic experiment. A higher concentration of citrate in leaves in comparison to stems was inconsistent with results for *R*. *obtusifolius* published by Miyagi et al. [34]—i.e., concentration of citrate was higher in stems. Discrepancies between concentrations of citrate in organs can be connected with the age of *R*. *obtusifolius* plants—i.e., 5-week-old plants have more citrate in stems and 6-month-old plants have more citrate in leaves. Citrate, oxalate, tartrate, and malate can create strong or moderate organic-Al complexes in organs [48,53]. Therefore we can speculate that these LMWOAs can partially contribute to the protection of *R*. *obtusifolius* against internal Al toxicity (i.e., mechanism of internal detoxification of Al known in plants that accumulate Al) [7,24,30,51].

Liming and application of superphosphate has an ambiguous effect on the concentration of LMWOAs in organs. We can thus conclude that our tested additives are not able to unambiguously alter the distribution of LMWOAs between organs of *R*. *obtusifolius* neither in slightly acidic nor in alkaline soils.

## Conclusions

Reducible Al fraction represented by the CA and OA solution agents can serve as simulating exudation of organic acids by *R*. *obtusifolius* and play an important role in potential Al mobility and availability for these plants mainly in alkaline soils.

Less or non-toxic exchangeable and water soluble monovalent Al cations are dominant soluble Al forms in slightly acidic and alkaline soils with pH ranging from 5.8 to 7.2.

*Rumex obtusifolius* is an Al-hyperaccumulator but only in untreated alkaline soils. We can speculate that mechanism for detoxification of Al is affected by different soil chemical properties. It is possible to change distribution of total Al in plant organs by the manipulation of Al availability using lime and superphosphate in tested soils. Restricted transport of total Al from belowground organs into leaves was recorded in both untreated soils and in superphosphate-treated slightly acidic soil. On the other hand, elevated transport of total Al from belowground organs into leaves was recorded in both lime-treated soils and in superphosphate-treated alkaline soil. Therefore, we can speculate that sufficient amount of Ca available from soil solution as well as from superphosphate can change distribution of total Al in plants of *R*. *obtusifolius*.

The highest concentration of Al was recorded in leaf infusion, followed by stem and belowground organ infusions as a result of Al release from weak stability Al-organic complexes. Concentrations of LMWOAs were not affected by different soil chemical properties but were greatly affected by plant organs. In belowground organs more citrate, maleate, and tartrate were recorded; in stems there was more malate, and in leaves higher concentrations of acetate, formate, lactate, propionate, and oxalate were recorded. Citrate and tartrate create strong organic-Al complexes in belowground organs and conversely oxalate creates strong organic-Al complexes in leaves. The distribution of LMWOAs in plant organs can play a crucial role in internal Al tolerance but the used methods did not allow us to distinguish the proportion of total Al-organic complexes to the free organic acids. Therefore the future research should be focused on this issue.
